# Mathematical modeling of quorum sensing dynamics in batch culture of luminescent bacterium Photobacterium phosphoreum 1889

**DOI:** 10.18699/VJGB-23-100

**Published:** 2023-12

**Authors:** S.I. Bartsev, A.B. Sarangova

**Affiliations:** Institute of Biophysics of the Siberian Branch of the Russian Academy of Sciences, Federal Research Center “Krasnoyarsk Science Center SB RAS”, Krasnoyarsk, Russia Siberian Federal University, Krasnoyarsk, Russia; Siberian Federal University, Krasnoyarsk, Russia

**Keywords:** quorum sensing, mathematical model, luminescent bacteria, кворум-эффект, математическая модель, люминесцентные бактерии

## Abstract

At the beginning of the paper, the level of necessary phenomenology of complex models is discussed. When working with complex systems, which of course include living organisms and ecological systems, it is necessary to use a phenomenological description. An illustration of the phenomenological approach is given, which captures the most significant general principles or patterns of interactions; the specific values of the parameters cannot be calculated from the first principles, but are determined empirically. An appropriate interpretation is also chosen empirically and pragmatically. However, in order to simulate a wider range of situations, it becomes necessary to lower the level of phenomenology, switch to a more detailed description of the system, introducing interaction between selected elements of the system. The requirements for a system model combining ecological, metabolic and genetic levels of cell culture description are formulated. A mathematical model of quorum sensing dynamics during the growth of batch culture of luminescent bacteria at different concentrations of the nutrient substrate has been developed. The model contains four blocks describing ecological, energy, quorum and luminescent aspects of bacterial culture growth. The model demonstrated good agreement with the experimental data obtained. When analyzing the model, three oddities in the behavior of the culture were noted, which presumably can change the idea of some processes taking place during the development of a culture of luminescent bacteria. The results obtained suggest the presence of some additional control system for the luminescent reaction via the synthesis pathways of FMN · Н2 or aliphatic aldehyde. In this case, the generalized description of the contribution of energy metabolism to luminescence only through ATP is too strong a simplification. As a result of comparing the model dynamics with the experiment, a discrepancy arose between the concentration of the substrate (peptone) measured in the experiment and its effective influence on the bacterial population growth. This discrepancy seems to indicate peptone is not the leading substrate, and growth is limited by nutrients contained in the yeast extract, the concentration of which did not change in these experiments. The discrepancies noted between the expectations and the results of experimental data processing, together with the assumptions about the causes of these discrepancies, set the direction for further experimental and theoretical studies of quorum sensing mechanisms in a culture of luminescent bacteria

## Abstract (ru)

В начале статьи обсуждается уровень необходимой феноменологичности сложных моделей. При работе со сложными системами, к которым, безусловно, относятся живые организмы и экологические системы, с необходимостью приходится использовать феноменологическое описание. Приведена иллюстрация феноменологического подхода, который ухватывает наиболее существенные даже не закономерности, а общие принципы или паттерны взаимодействий, причем конкретные значения параметров не могут быть вычислены из первых принципов, а определяются эмпирически. Также эмпирически и прагматически выбирается соответствующая интерпретация. Однако для моделирования более широкого круга ситуаций возникает необходимость понижать уровень феноменологии, переходить на более детальное описание системы, вводя взаимодействие между выделенными элементами системы. Формулируются требования к модели системы, совмещающей экологический, метаболический и генетический уровни описания клеточной культуры. Разработана математическая модель динамики кворум-эффекта в процессе роста накопительной культуры люминесцентных бактерий при разных концентрациях питательного субстрата. Модель содержит четыре блока, описывающие экологический, энергетический, кворумный и люминесцентный аспекты развития культуры. Модель продемонстрировала хорошее соответствие экспериментальным данным, полученным в ходе выполнения работы. При анализе модели отмечены три странности в поведении культуры, которые, предположительно, могут изменить представление о некоторых процессах, имеющих место при развитии культуры люминесцентных бактерий. Полученные результаты позволяют предположить наличие некоторой дополнительной системы контроля люминесцентной реакции через пути синтеза ФМН · Н2 или алифатического альдегида. В этом случае обобщенное описание вклада энергетического метаболизма в люминесценцию только через АТФ является слишком сильным упрощением. В результате анализа результатов сопоставления модельной динамики с экспериментом возникло расхождение между измеряемой в эксперименте концентрацией субстрата (пептона) и его эффективным действием на рост популяции бактерий. Это расхождение, по-видимому, указывает на то, что пептон не является ведущим субстратом и рост лимитируют биогены, содержащиеся в дрожжевом экстракте, концентрация которого в этих экспериментах не изменялась. Отмеченные расхождения между ожиданиями и результатами обработки экспериментальных данных вместе с предположениями о причинах этих расхождений задают направление дальнейших экспериментальных и теоретических исследований механизмов кворум-эффекта в культуре люминесцентных бактерий.

## Introduction

When working with complex systems, which of course include living organisms and ecological systems, it is necessary to use a phenomenological description. One of the widely used examples of a phenomenological description of a population is the Verhulst equation. Despite the fact that formally the population equations can be used only near the threshold of population survival (Gorban et al., 1982), this equation describes the dynamics of various processes quite well: the batch culture of microorganisms, the spread of an epidemic under constant conditions, population growth after invasion and the dynamics of sales under conditions of limited market capacity. Apparently this is due to the fact that at the final stage of the process, when the value of the variable approaches the carrying capacity, the specific growth rate approaches 0, which, in fact, corresponds to the approach to the threshold of survival.

Several versions of the Verhulst equation can be written, corresponding to different interpretations. Consider, for example, two of them:

**Formula. 1.**
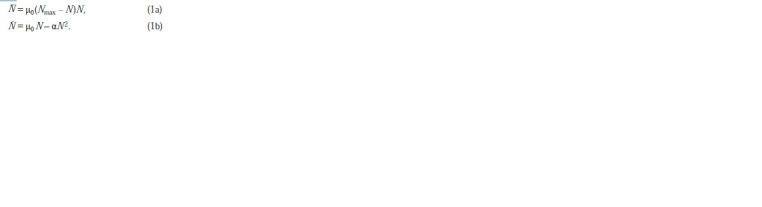
Formula 1

In the first version, Nmax is called the carrying capacity, understood as the maximum population size that can exist under given conditions, and the product of μ0 Nmax is the specific rate of population growth at a population size close to zero. The carrying capacity phenomenologically includes all kinds of factors limiting the growth of the population: substrate inhibition, inhibition by metabolites, limited plant growth area. This option corresponds well to the interpretation of plant or microbial population growth.

In the second version, μ0 is the specific growth rate, α is a coefficient describing intraspecific competition, which can be realized by different mechanisms – competition for food and/or displacement from the hunting territory and direct collisions of individuals. This interpretation seems to be more appropriate for animals.

These examples are given to illustrate the phenomenological approach, which captures the most significant, not even regularities, but general principles or patterns of interactions, and the specific values of the parameters cannot be calculated from the first principles, but are determined empirically. The appropriate interpretation is also chosen empirically and pragmatically.

However, to model a wider range of situations, there is a need to lower the level of phenomenology, move to a more detailed description of the system, introducing interaction between selected elements of a system. For example, there are cases when the Verhulst equation does not describe the dynamics of a batch culture accurately enough. In this case, it is necessary, for example, to take into account substrate dynamics and introduce substrate inhibition of culture growth. At the same time, we still remain at a very high level of phenomenology, continuing to describe the dependence of culture growth using the Monod formula and its various modifications and complications, reducing the entire metabolism of a cell or multicellular organism to one key enzymatic reaction.

The need to lower the level of phenomenology arises when the researcher encounters phenomena that do not fit into the existing model. In this case, it is often necessary to move to the level of genetic and/or metabolic regulation of cellular processes. One such example that requires reducing the phenomenological nature of the models used is quorum sensing (QS) (Miller, Bassler, 2001). It is noteworthy and symbolic that QS, which is a manifestation of molecular-level events at the population level, was discovered in luminescent bacteria, the luminescence of which is a natural indicator of the current state of cellular metabolism (Nealson et al., 1970).

The quorum sensing is the expression of certain genes being triggered when a certain threshold population density is reached. At the bacterial level, this effect is based on the synthesis and release into the external environment of signal molecules (autoinducers), the concentration of which varies depending on the number of surrounding cells, and, when a certain threshold concentration is exceeded, the expression of certain genes is triggered. Since QS occurs in a fairly wide range of organisms (for example, insects (Anstey et al., 2009) and fish (Makris et al., 2009)), its study seems quite important in itself. In addition, identifying the patterns of manifestation of QS and its prediction is important for the microbiological synthesis of products triggered by this effect. An example of such a product is bacterial luciferase, which is used for laboratory and rapid toxicological biotests. At the same time, luminescent bacteria are a convenient tool for studying QS, since luminescence is a natural function of cells, which makes it possible to study the process on native cells without the introduction of special fluorescent dyes and without stimulating fluorescence. The evolutionary meaning of QS in luminescent bacteria is explained within the framework of the hypothesis that the selection mechanism is associated with spread and reproduction of bacteria (Nealson, Hastings, 1979). As marine enterobacteria, luminescent bacteria growing on a substrate (the surface of dead organisms or fecal pellets), if the culture density is sufficient, can produce enough light to attract organisms to consume them, thereby ensuring the circulation of bacteria through the intestinal tracts of sea animals.

The purpose of this work is to develop a mathematical model and its software implementation for the analysis of experimental data on QS in batch culture of luminescent bacteria. To specify the requirements for the model, we will formulate a kind of technical specification (TS) for the model being developed. Firstly, the model must describe the dynamics of bacterial growth in batch culture; secondly, it must describe the dynamics of the luminescence of a bacterial culture, which is regulated by QS, i. e. events at the molecular level; thirdly, the model should be as simple as possible for the simple reason that a complex model contains a large number of parameters with unknown values, i. e. we follow the paradigm that the fewer fitting parameters there are in a model describing complex processes, the more it reflects the essence of the processes being modeled.

The third point of our conditional TS mentions the complexity of the model, and since this point demands the simplicity of the model being created, at least a brief discussion of this term is required. Unfortunately, there is no universal definition of complexity; this is evidenced by the huge (>40) number of existing definitions of complexity (Edmonds, 1999). The peculiarities of applying this term to the description of evolving living systems make it possible to narrow down the set of possible definitions (Bartsev, Bartseva, 2010). In the case of mathematical models constructed as systems of ordinary differential equations (ODEs), often used to describe the chemical (biochemical) kinetics and dynamics of ecological systems, a natural (or at least widely used) indicator of complexity is the number of differential equations in the system. Apparently, it is not for nothing that methods that make it possible to reduce the dimension of an ODE system, for example, by selecting a subsystem of fast motions and applying Tikhonov’s theorem (Romanovsky et al., 1984), are called methods for simplifying systems of kinetic equations.

True, the question remains about the complexity of the equations themselves, or rather, their right-hand sides. It is obvious that functions including a larger number (so to speak) of nonlinearities, for example, terms with large powers in a fractional rational function, can provide more diverse behavior. Apossible quantitative approach to assessing the complexity of ODE systems, taking into account the degree of nonlinearity of the right-hand sides, can be based on Korzukhin’s theorem (Jabotinsky, 1974). It states that for a system with nonlinear right-hand sides, a system of chemical kinetics equations (containing terms that describe reactions no higher than second order) can be constructed so that the behavior of some of the variables of the new system will coincide with the behavior of the variables of the original one. The number of equations of the second, expanded system could serve as a measure of the complexity of the model, taking into account the degree of nonlinearity of the right-hand sides used. Since our task is not to obtain an accurate estimate of the model complexity, but only to construct the simplest possible model that provides an adequate description of the real system, we will simply minimize the number of differential equations of the model and simultaneously use the minimum degrees of variables in there right-hand sides.

## Materials and methods

Experimental part. The object of the study are luminous bacteria Photobacterium phosphoreum 1889, from the collection of the Institute of Biophysics SB RAS. Bacterial growth was assessed by measuring optical density at 660 nm on an Agilent Cary 60 spectrophotometer. To measure the bioluminescence of the reaction mixture Promega GloMax 20/20 Luminometer (USA) was used. The bacteria were grown in a liquid medium for marine bacteria (g/l): NaCl – 28.5, KCl – 0.5, CaCl2 – 0.5, MgCl2 – 4.5, yeast extract – 1, peptone – 10; pH 7.6.

Mathematical model. The bioluminescent system of bacteria has been very well studied (Brodl et al., 2018), the enzymes expressed jointly when QS is triggered are known, and the pathways for the synthesis of substrates for the luminescent reaction are quite well studied. For us, in order not to dive into the details of the kinetics of the multienzyme system, the following is important: the direct substrates of the luminescent reaction are reduced flavin mononucleotide (FMN·H2), long-chain aliphatic aldehyde – tetradecanal and molecular oxygen. The flavin is reduced by the enzyme NADH:FMN oxidoreductase, and the aldehyde is synthesized by the ATP-consuming fatty acid reductase enzyme complex. Thus, the luminescent reaction is directly related to the energy metabolism of the cell, and its luminescence depends not only on the amount of luciferase in the cell, but also on the state of its energy metabolism.

Consequently, already at the level of describing crop growth, an assessment of the state of its energy metabolism must be included in the model. The properties of the multienzyme system of energy metabolism were studied in detail in the almost forgotten (judging by the citation statistics from ResearchGate) work of E.E. Selkov, which is part of a collective monograph (Ivanitsky et al., 1978). One of the most important properties of energy metabolism is maintaining a constant intracellular ATP concentration within a wide range of consumer load to ensure decoupling (relative independence) of intracellular energy consumers. In his work, the case of a constant rate of substrate supply with varying load (activity of generalized ATPase) was considered. In this model, it is necessary to take into account both the change in the rate of substrate supply (in our case, we will consider its concentration in the medium) and changes in ATPase activity associated with different phases of culture growth. Here the entire Selkov’s model, in accordance with TS-3, will not be reproduced, but some of his ideas will be applied.

When writing a model that, on the one hand, describes the variables characterizing a bacterial culture – substrate concentration and biomass density in the flask, and, on the other hand, should describe the average intracellular ATP concentration, it is necessary to coordinate the rates of the processes. If we designate the volume of the flask as Vc , and the total volume of bacterial cells as Vb , then between the rates of processes expressed in concentrations per unit of time – vc and vb, respectively, due to the conservation law, the following relation must be satisfied: vc·Vc = vb·Vb , where the right and left sides of the equality describe the rate of change in the mass of the reagent. It follows that the rates of intracellular processes must exceed the (concentration) rates of the same processes by Vc /Vb times and we will have a system with different characteristic times of change in variables. Let us denote the ratio Vb /Vc as a small parameter ε0.

Taking into account the above, the “ecological part” of the model can be written as follows:

**Formula. 2.**
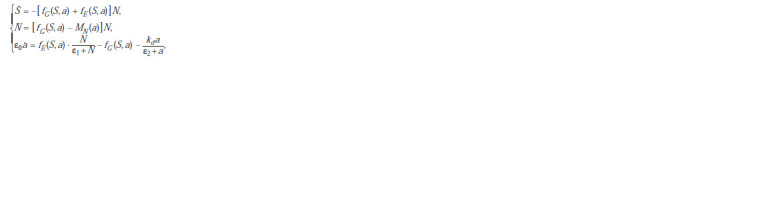
Formula 2

where S is the concentration of the nutrient substrate; N is the bacterial biomass; a is the average intracellular concentration of ATP in the cells of a bacterial culture.

In this case, the function

**Formula. 3.**
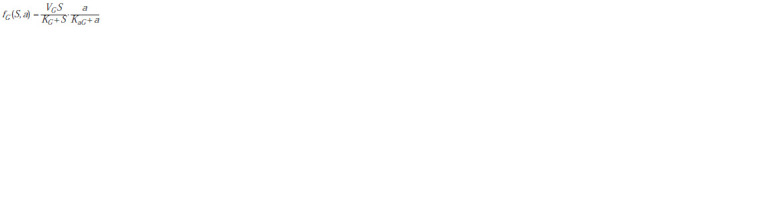
Formula3

describes the ATP-dependent synthesis of biomass, the function

**Formula. 4.**
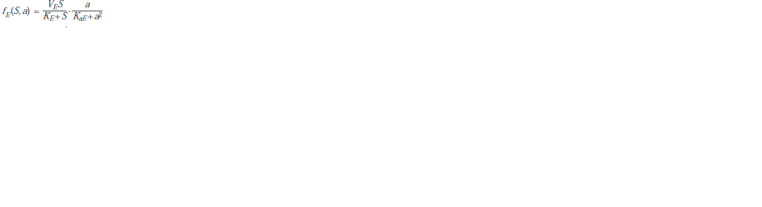
Formula4

describes the production of ATP, the expression

**Formula. 5.**
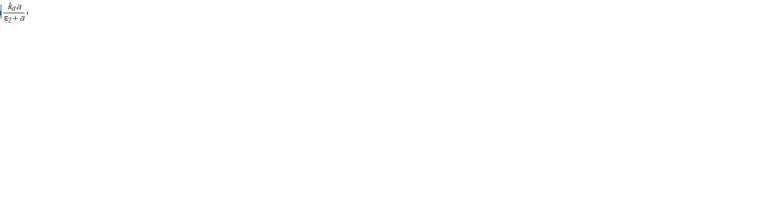
Formula5

describes the activity of the generalized ATPase, and the function

**Formula. 6.**
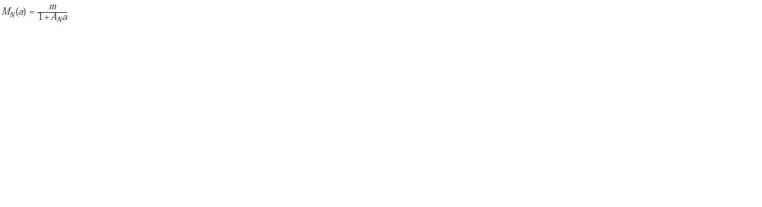
Formula6

describes the intensity of bacterial death, depending on the intracellular ATP concentration.

As one can see, in this model the generalized activities of anabolic and catabolic pathways are described by separate functions, so there is no need to specifically introduce the so-called economic coefficient; moreover, the ratio of the rates of biomass synthesis and organic oxidation may change during crop growth. The type of function fE (S, a), or more precisely its part, describing the dependence of the activity of ATP synthesis on its concentration, was chosen in accordance with Selkov’s model (Ivanitsky et al., 1978). The last term in the equation describing the ATP concentration represents the contribution of the generalized ATPase, i. e. the totality of all basic processes in a cell. At small values of the coefficient ε2 ATPase activity will change little over a wide range of ATP concentrations, and only at low values a drop in ATPase activity will be observed, which seems natural

The presence of a small parameter in the third equation makes the ATP concentration a fast variable and allows us to study the properties of this equation separately from other variables, assuming the remaining (ecological) variables are constants (Romanovsky et al., 1984). We will not do a complete analysis of the stability of this equation due to its cumbersomeness; it is enough for us, in accordance with the technical specifications, to check the possibility of the existence of a stable quasi-stationary state of a given dynamic system and evaluate the dependence of its stability on the values of environmental variables.

**Fig. 1.**
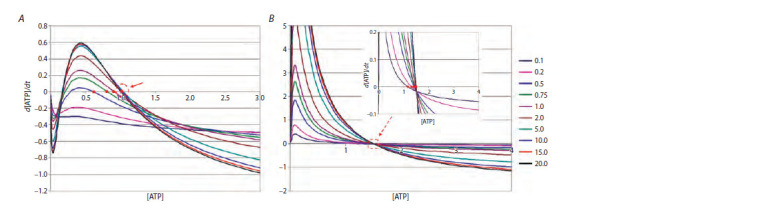
Dependence of the rate of ATP concentration on its concentration at different substrate concentrations (shown on the right) at different sets of parameter values highlights a group of indistinguishable stationary states at different values of S. Case B: there is one stable and one unstable zero stationary state in the system. The red circles show stable stationary states at different substrate concentrations. Appropriate parameter sets for cases А: Vg = 1.22, Kg = 1.94, Ka = 0.01, Ve = 2, Ke = 1, Kae = 0.2, kd = 0.5, ε2 = 0.05 and B: Vg = 2.18, Kg = 4, Ka = 0.004, Ve = 3.299, Ke = 4, Kae = 0.008, kd = 0.026, ε2 = 0.85.

From Figure 1 we can see that depending on the set of parameters the system can have: (A) one stable zero stationary state, or three stationary states depending on the substrate concentration S; (B) one unstable zero and one stable stationary state for any values of concentration S. Since at this stage we are not concerned with the exact correspondence of the parameters of the cell energy system model to real data, we will follow Selkov’s approach and the stated technical specifications. It means we will choose an option, on the one hand, providing the cell with stable satisfaction of its energy needs, and on the other, doing this in the simplest way. From Figure 1 it is clear that this requirement is met by a set of parameters that generates the dependencies presented in the sub-figure (B).

It can be seen from the figure that at certain parameter values there is a range of changes in the ATP concentration, in which the rate of ATP synthesis is positive, which leads to an increase in its concentration until the concentration falls into the region with a negative rate value, which ensures the existence of a stable stationary state.

Having ensured, relatively speaking, the vital activity of the cell, we can move to constructing a model of QS. Let us consider the QS model (Williams et al., 2008), which was subsequently used in a number of works by other authors (Melke et al., 2010; Djezzar et al., 2019). According to this model, the autoinducer AHL (A) and the receptor LuxR (R) form a dimerized complex that regulates the production of both R and A. In addition, there is a nonzero, basal, inducer concentration-independent synthesis of LuxR. The model looks like this:

**Formula. 7.**
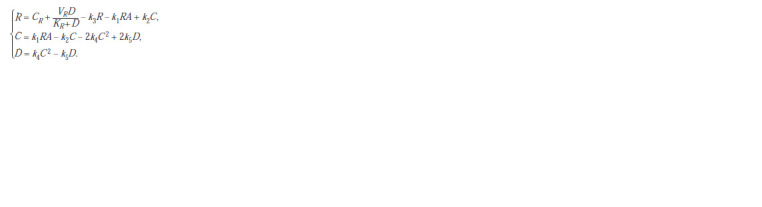
Formula7

In this system, the first equation describes the rate of change in the LuxR concentration, which positively depends on the sum of the basal (CR) and autoinduced synthesis rates. The latter is proportional to the probability of transcription initiation controlled by binding the (LuxR-A)2 (D) complex to the corresponding binding site in the regulatory sequence of the operon. The second and third equations describe the formation of the LuxR-A complex (C) followed by the formation of the dimeric complex (LuxR-A)2 (D).

Following (Williams et al., 2008) and TS-3, we will assume the existence of a quasi-stationary state for variables C and D. Then, the equation describing the behavior of LuxR at the concentration of the autoinducer considered as an external parameter has the form:

**Formula. 8.**
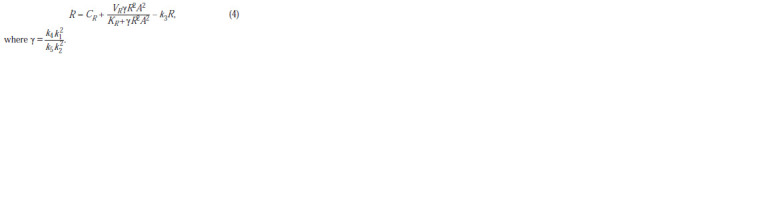
Formula8

**Fig. 2.**
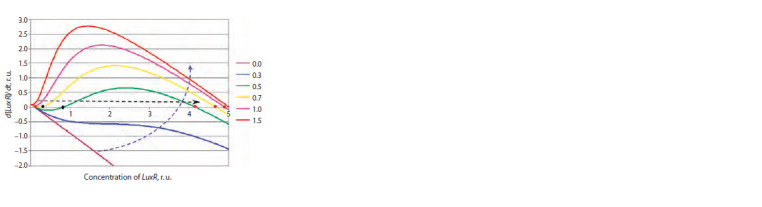
Dependence of the rate of change in the concentration of LuxR on its concentration at different concentrations of the auto-inductor shown on the right. The red circles show stable stationary states at different concentrations of the auto-inductor, the black ones show unstable states. The curved dashed arrow indicates the direction of change in the concentration of the auto-inductor. The straight dashed arrow shows the direction of switching to the new state.

To analyze the properties of this equation, one can apply the technique used for the third equation of system (2), that is, consider it in coordinates (R, dR/dt) at different concentrations of the autoinducer, which is a simple matter (Fig. 2). It can be seen from the figure that at zero and low substrate concentrations, only one stable stationary state can exist, corresponding to a low LuxR concentration. As the concentration of the autoinducer increases, two more stationary states appear – stable and unstable, but the system cannot voluntarily switch to a state with a high level of LuxR expression. With a further increase in the concentration of the autoindicator, the left knee of the curve leaves the negative half-plane, which leads to the disappearance of the unstable and stable states and the system quickly transitions to a state with a high concentration of LuxR.

The switching process can be shown more clearly if we assume that a quasi-stationary state of the system described by equation (4) is realized. In this case, one can either apply plotting implicitly defined functions in computer algebra systems like Maxima, or, by setting the right side equal to 0, obtain an expression for the explicit function

**Formula. 9.**
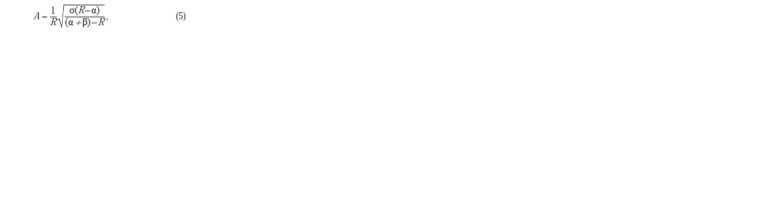
Formula9

where σ = KRγ; α = CR/k3; β = VR/k3. In this case, the condition α <R <α + β must be satisfied

**Fig. 3.**
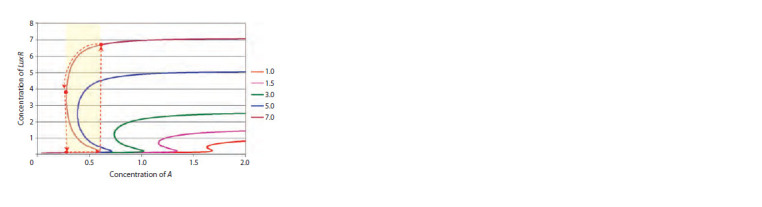
Stationary curves showing the dependence of the stationary LuxR concentration on the concentration of the auto-inductor at α = 0.1, σ = 1 and different values of the parameter β (right).

For clarity, one can tabulate (5) as a regular function in Excel, and then flip the coordinates – make (A, R) (Fig. 3). The figure clearly shows that when a certain threshold concentration of А(β) is exceeded, a sharp transition to a state of high level of LuxR expression occurs, and hysteresis can be observed in the system, which under natural conditions can be observed when bacterial growth is inhibited and the autoinducer is gradually destroyed.

After running the QS model and tentatively estimating the values of the parameters that are necessary to implement QS, we will return to building the total model. Using the well-known model discussed above, we will slightly modify it to ensure its conceptual unity, namely, we will make the intensive synthesis of LuxR energy-dependent. In this case, we will leave the background synthesis of the autoinducer and LuxR conditionally energy-independent, considering that the costs of their synthesis are included in the activity of the generalized ATPase (2):

**Formula. 10.**
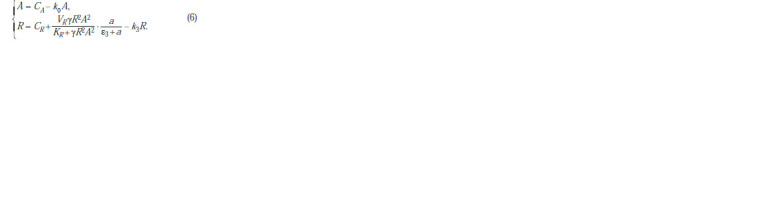
Formula10

Looking ahead, we can say that the use of a more complex equation, assuming that simultaneously with the synthesis of LuxR, the synthesis of the autoinducer is intensified, as was done in the model (Melke et al., 2010), turned out to be unnecessary to describe the experimental data. In addition, for simplicity, it is assumed that the concentration of the autoinducer in the medium and in the cell coincide, which makes it possible to avoid selecting a small parameter. As a result, our model, combining environmental and intracellular molecular processes, looks like this:

**Formula. 11.**
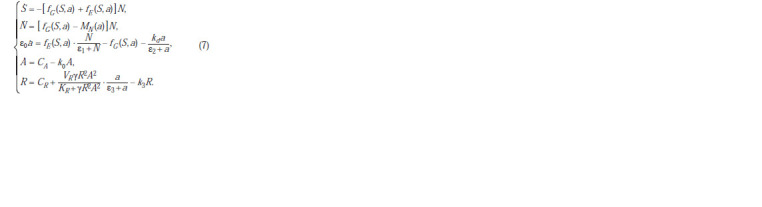
Formula11

Let’s start constructing the final luminescent block of the model. First, let us assume that the synthesis of luciferase occurs in parallel with the synthesis of LuxR and is also energy dependent. In addition, we will take into account the energyindependent process of luciferase inactivation. However, in this experiment we record not the amount of luciferase in the culture, but the intensity of luminescence. As mentioned above, to ensure luminescence, NADH and ATP must come from the cell. It is possible to take these flows into account separately, but it hardly makes sense, since the activity of the cytochrome chain that produces ATP depends on the presence of NADH. Thus, since these processes are closely related and the presence of ATP means the presence of NADH, in the model (following TS-3) we will consider the dependence of luminescence only on ATP. As a result, we obtain a general model of the system under consideration:

**Formula. 12.**
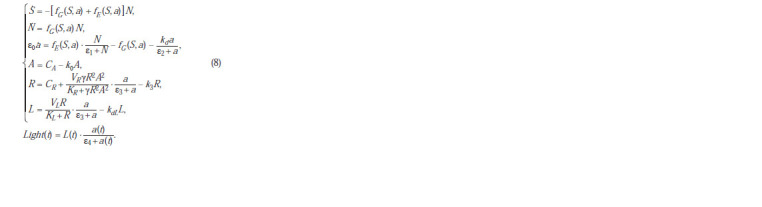
Formula12

The difference between the ecological part of this model and (2) is that since the experiment considers batch culture from inoculation to the logarithmic growth phase inclusively, without considering the stationary phase and the death phase, the mortality of bacteria cannot be taken into account in this experiment.

The mathematical model was implemented in the open source environment SciLab 6.1. To determine the parameters of the mathematical model from experimental data, the Nelder–Mead method was used, the code of which is included in the accompanying software examples.

## Results

**Fig. 4.**
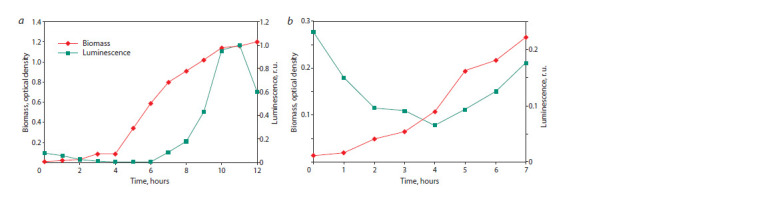
Dynamics of biomass growth and luminescence of Photobacterium phosphoreum 1889 culture on a standard medium (a) and on a poor medium (b).

Test experiments carried out on the prescribed rich (10 g/l peptone) and poor media (0.1 g/l peptone) showed the presence of QS in both cases. The biomass and luminescence dynamics curves are shown in Figure 4.

Simply examining the obtained curves, without any model, one can see (see Fig. 4, a) that before the start of QS (within 6 hours of cultivation) there is a gradual decrease in the intensity of luminescence produced by luciferase brought with the inoculum. At the same time, after reaching the maximum of the glow (~11 hours), a sharp decrease in the intensity of the glow is observed. It is almost obvious that such a decline cannot be associated with inactivation of luciferase, which would require the assumption of the existence of a special system that destroys luciferase immediately after synthesis, and even under conditions of energy starvation. Apparently, it was the drop in the concentrations of NADH and ATP at the final stage of the logarithmic phase of culture growth that caused this drop in luminescence. At the same time, the slow decrease in luminescence intensity, which took place under conditions of excess substrate and intensive energy metabolism, demonstrates the process of inactivation of luciferase, or more precisely the complex of enzymes that serve the luminescence of bacteria.

At the same time, the dynamics of cultural luminescence in a poor environment (see Fig. 4, b) raises interesting questions. It can be seen that after 7 hours of cultivation, the biomass of bacteria reached approximately more than a third of the biomass achieved by bacteria during the same time in the rich medium. At the same time, the growth rate of the culture, although not very high, was approximately constant throughout the entire period under consideration, which cannot be said about the other experiment. The obvious acceleration of culture growth in a rich medium after 4 hours of growth may indicate substrate inhibition at given substrate concentrations.

It is interesting that in the poor medium QS began 2 hours earlier than in the rich medium. It is possible that substrate inhibition has this effect, but this issue requires further research and more experimental material. At this stage, our objective is to develop an adequate model that satisfies the technical specifications stated at the beginning of the article, and to preliminarily test the adequacy of this model using the available experimental data.

**Fig. 5.**
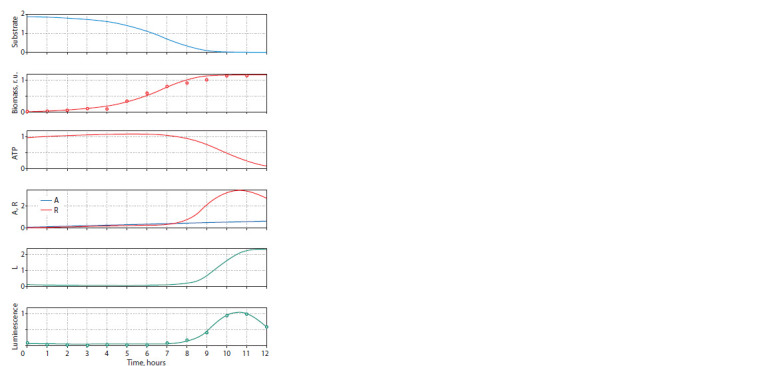
Model and real dynamics of variables in the culture of luminescent bacteria. Circles indicate experimental data.

**Fig. 6.**
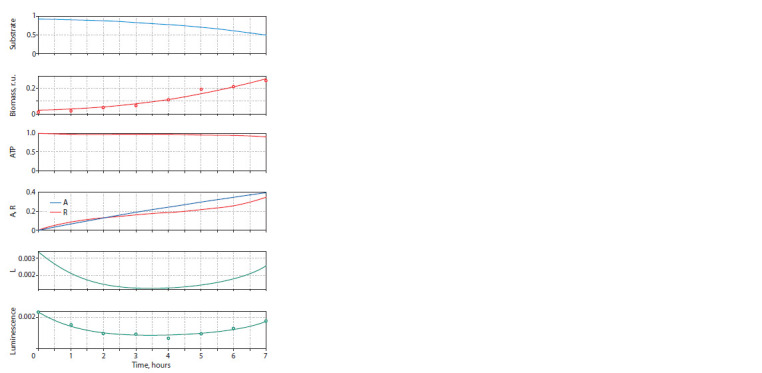
Model and real dynamics of variables in the culture of fluorescent bacteria in a poor environment Circles indicate experimental data.

The results of computational modeling are shown in Figures 5 and 6. The adjustment of the model parameters took place in two stages – first, the ecological part was adjusted, describing the dynamics of the biomass of the bacterial culture, substrate concentration and the average intracellular ATP concentration. The results are shown in the top three graphs of the presented figures. It should be noted that there is fairly good agreement between the model curve describing the dynamics of biomass and the experimental points. As additional calculations have shown, neither the Verhulst model nor the introduction of the substrate inhibition factor into the model provides an improved description. Two options are possible: either the observed discrepancy is of a statistical nature, or there is a mechanism in the system that accelerates growth after reaching a certain threshold. Further analysis will require additional experiments, which are planned.

It should be noted the expected behavior of the ATP concentration, which, as can be seen from Figure 1, should undergo minor changes when the substrate concentration varies in a certain interval and change quite sharply when leaving this interval.

At the second stage, the part of the model describing QS and luminescence was adjusted, and data on luminescence dynamics were used as reference data. At the same time, the “ecological and energy” parameters of the model did not change.

Figure 5 shows the model dynamics of the autoinducer and LuxR, as well as the dynamics of the amount of luciferase, which follows the dynamics of LuxR expression. It is important to note that the model clearly describes the slow decrease in luminescence at the initial stage of culture growth and its rapid decrease at the final stage, which differs in rate from the decrease in the amount of luciferase, which represents the energy state of the cells.

In the case of modeling the behavior of a culture in a poor environment (see Fig. 6), the following point can be noted. It is important that a model containing a large number of adjustable parameters is capable of describing various variants of dynamics, and the question is how well these parameters correspond to biological ideas about the system under study. Looking at the graphs in Figure 6, one can note good agreement between the model curves and the experimental data. Let us compare in the “Discussion” section the changes in the constants that were made by the system for adjusting parameters when describing the growth of a culture in a poor environment. In this case, the values of the model parameters common for the two cases are as follows: Vg = 2.18, Kg = 3.99, Ka = 0.0033, Ve = 3.30, Ke = 4.02, Kae = 0.008, a0 = 1.40, kd = 0.0315, k0 = 0.082, VR = 1.50, CA = 0.14, CR = 0.011, k3 = 0.057, γ = 0.331, KR = 0.06, KL = 0.17, ε0 = 0.01, ε1 = 0.001, ε2 = 1.54, ε3 = 0.39, ε4 = 3.34.

## Discussion

From the Table it can be seen that it was not necessary to change a very large number of parameters in order to obtain a good description of the dynamics of culture in both experiments. Note that the change in S0 is expected; another thing is that the almost twofold decrease in S0 in the model is in poor agreement with a hundredfold decrease in the peptone concentration in the medium. This discrepancy can be tentatively explained by the fact that, apparently, peptone is not the leading substrate and growth is limited by nutrients contained in the yeast extract, the concentration of which did not change in these experiments.

**Table 1.**
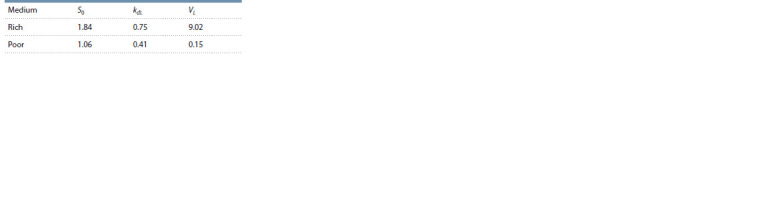
Comparison of model parameters for two types of nutrient medium

Questions arise regarding changes in the other two parameters. Such a significant (60 times!) drop in the VL constant can only be explained by the presence of some additional system for controlling the luminescent reaction through the synthesis pathways of FMN ·H2 or aliphatic aldehyde. In this case, a generalized description of the contribution of energy metabolism only through ATP is too strong a simplification.

The almost twofold decrease in the kdL constant during growth in a poor medium is also difficult to explain. It is premature to build hypotheses on this matter; we can return to the issue after obtaining additional experimental data.

The noted discrepancies between expectations and the results of processing experimental data, together with assumptions about the courses of these discrepancies, set the direction for further experimental and theoretical studies of the mechanisms of QS in the culture of luminescent bacteria.

## Conclusion

The results of a comparison of the model built within the framework of the presented logic and experimental data show that the proposed model generally satisfies the conditional technical specifications that were formulated in the Introduction. Indeed, (1) the model quite satisfactorily describes the dynamics of bacterial biomass in batch culture, (2) the model clearly describes the dynamics of the luminescence of a bacterial culture, which is regulated by QS.

But regarding the third requirement of the technical specifications about the maximum simplicity of the model, it is difficult to give a final assessment. On the one hand, it is possible that this model can be simplified to describe the behavior of bacterial cultures under conditions close to the conditions of the experiments considered. On the other hand, working with the model (selection of parameters) made us feel that this model is not robust enough with respect to the variation of parameters. This was manifested, in particular, in the fact that the Nelder–Mead method, like any local search method, quite often finds the nearest minimum of the goal function, which corresponds to the values of parameters that are distantly related to biological meaning (the tendency of the Monod constant to 0). It is possible that a model, in which the semantic blocks (ecological, energy, quorum, luminescent) will be more articulated, more autonomous, in line with the ideas of E.E. Selkov, will be resistant to external and internal disturbances, almost like a living being.

## Conflict of interest

The authors declare no conflict of interest.

## References

Anstey M.L., Rogers S.M., Ott S.R., Burrows M., Simpson S.J. Serotonin
mediates behavioral gregarization underlying swarm formation
in desert locusts. Science. 2009;323(5914):627-630. DOI
10.1126/science.1165939

Bartsev S.I., Bartseva O.D. Heuristic Neural Network Models in Biophysics:
Application to the problem of structure–function mapping.
Krasnoyarsk: Siberian Federal University Publ., 2010 (in Russian)

Brodl E., Winkler A., Macheroux P. Molecular mechanisms of bacterial
bioluminescence. Comput. Struct. Biotechnol. J. 2018;16:551-
564. DOI 10.1016/j.csbj.2018.11.003

Djezzar N., Pérez I.F., Djedi N., Duthen Y. A computational multiagent
model of bioluminescent bacteria for the emergence of self-sustainable
and self-maintaining artificial wireless networks. Informatica.
2019;43(3):395-408. DOI 10.31449/inf.v43i3.2381

Edmonds B. Syntactic Measures of Complexity. Doctoral Thesis. Manchester,
UK: Univ. of Manchester, 1999.

Gorban A.N., Okhonin V.A., Sadovskiy M.G., Khlebopros R.G. The
simplest equation of mathematical ecology. Preprint of the Sukachev
Forest and Timber Institute, Siberian Branch of the USSR Academy
of Sciences, 1982 (in Russian)

Ivanitsky G.R., Krinsky V.I., Selkov E.E. Mathematical Biophysics of
the Cell. Moscow: Nauka Publ., 1978 (in Russian)

Jabotinsky A.M. Concentration Oscillations. Moscow: Nauka Publ.,
1974 (in Russian)

Makris N.C., Ratilal P., Jagannathan S., Gong Z., Andrews M., Bertsatos
I., Godø O.R., Nero R.W., Jech J.M. Critical population density
triggers rapid formation of vast oceanic fish shoals. Science.
2009;323(5922):1734-1737. DOI 10.1126/science.1169441

Melke P., Sahlin P., Levchenko A., Jӧnsson H. A cell-based model for
quorum sensing in heterogeneous bacterial colonies. PLoS Comput.
Biol. 2010;6(6):e1000819. DOI 10.1371/journal.pcbi.1000819

Miller M.B., Bassler B.L. Quorum sensing in bacteria. Annu. Rev. Microbiol.
2001;55(1):165-199. DOI 10.1146/annurev.micro.55.1.165

Nealson K.H., Hastings J.W. Bacterial bioluminescence: its control and
ecological significance. Microbiol. Rev. 1979;43(4):496-518. DOI
10.1128/mr.43.4.496-518.1979

Nealson K.H., Platt T., Hastings J.W. Cellular control of the synthesis
and activity of the bacterial luminescent system. J. Bacteriol. 1970;
104(1):313-322. DOI 10.1128/jb.104.1.313-322.1970

Romanovsky Yu.M., Stepanova N.V., Chernavsky D.S. Mathematical
Biophysics. Moscow: Nauka Publ., 1984 (in Russian)

Williams J.W., Cui X., Levchenko A., Stevens A.M. Robust and sensitive
control of a quorum-sensing circuit by two interlocked feedback
loops. Mol. Syst. Biol. 2008;4:234. DOI 10.1038/msb.2008.70

